# β-Globin *Sleeping Beauty* Transposon Reduces Red Blood Cell Sickling in a Patient-Derived CD34^+^-Based In Vitro Model

**DOI:** 10.1371/journal.pone.0080403

**Published:** 2013-11-18

**Authors:** Lucas M. Sjeklocha, Phillip Y.-P. Wong, John D. Belcher, Gregory M. Vercellotti, Clifford J. Steer

**Affiliations:** 1 Department of Medicine, Division of Gastroenterology, Hepatology and Nutrition, University of Minnesota Medical School, Minneapolis, Minnesota, United States of America; 2 Vascular Biology Center, University of Minnesota Medical School, Minneapolis, Minnesota, United States of America; 3 Department of Medicine, Division of Hematology, Oncology and Transplantation, University of Minnesota Medical School, Minneapolis, Minnesota, United States of America; 4 Department of Genetics, Cell Biology and Development, University of Minnesota, Minneapolis, Minnesota, United States of America; Emory University School of Medicine, United States of America

## Abstract

The ultimate goal of gene therapy for sickle cell anemia (SCA) is an improved phenotype for the patient. In this study, we utilized bone marrow from a sickle cell patient as a model of disease in an *in vitro* setting for the hyperactive *Sleeping Beauty* transposon gene therapy system. We demonstrated that mature sickle red blood cells containing hemoglobin-S and sickling in response to metabisulfite can be generated *in vitro* from SCA bone marrow. These cells showed the characteristic morphology and kinetics of hemoglobin-S polymerization, which we quantified using video microscopy and imaging cytometry. Using video assessment, we showed that delivery of an IHK-β^T87Q^ antisickling globin gene by *Sleeping Beauty* via nucleofection improves metrics of sickling, decreasing percent sickled from 53.2 ± 2.2% to 43.9 ± 2.0%, increasing the median time to sickling from 8.5 to 9.6 min and decreasing the maximum rate of sickling from 2.3 x 10^-3^ sickling cells/total cells/sec in controls to 1.26 x 10^-3^ sickling cells/total cells/sec in the IHK-β^T87Q^-globin group (*p* < 0.001). Using imaging cytometry, the percentage of elongated sickled cells decreased from 34.8 ± 4.5% to 29.5 ± 3.0% in control versus treated (*p* < 0.05). These results support the potential use of *Sleeping Beauty* as a clinical gene therapy vector and provide a useful tool for studying sickle red blood cells *in vitro.*

## Introduction

Sickle cell anemia (SCA) is famously known as the first ‘molecular disease’ [1]. It is caused by a single nucleotide change in the sixth codon of the β-globin gene, which produces hemoglobin (Hb) with a propensity to polymerize when deoxygenated, forming rigid fibers that distort red blood cell shape and lead to numerous pathologic sequelae [2,3]. The long understood cause of the disease belies a complex pathophysiology for which effective therapies for many patients have remained elusive, but it is the characteristic Hb polymerization, sickling, and loss of deformability of affected red cells which gives rise to the devastating consequences for the fifty thousand Americans and greater than three million people worldwide with SCA [2,3]. A limited number of effective therapies are presently available to patients, and the only definitive cure is a hematopoietic stem cell (HSC) transplant. SCA is an attractive candidate for gene therapy with many potential strategies, primarily by the delivery of a therapeutic globin gene to hematopoietic stem cells by viral or non-viral vectors [4].

The *Sleeping Beauty* transposon system (SB) is a non-viral means to deliver a potentially therapeutic transgene [5-7]. First developed more than a decade ago from transposon fossils in the salmonid genome, the system has undergone major improvements, and has demonstrated significant utility in modifying HSCs, most recently using a hyperactive variant termed SB100X [7-11]. The system delivers a transposon, a transgene flanked by a pair of inverted repeats, which is inserted into the genome at random TA-dinucleotides via the *SB* transposase that is co-delivered with the transposon. The favorable insertion profile of *SB* relative to viruses and the persistent issues of oncogenesis and clonal expansion in viral therapies make *SB* an attractive candidate to increase safety in integrating gene therapy vectors [12-17].

Recently, transplantation trials in humans and numerous animal studies have shown that complete correction or replacement of the hematopoietic stem cell pool or correction of the β^S^ point mutation itself are not required to provide therapeutic benefits [18-20]. Given these encouraging trials and the natural history of sickle cell trait, we sought to introduce a competing anti-sickling globin gene to HSCs to test for potential phenotypic correction.

The most definitive studies of correction in terms of sickling phenotype have been in animals; however, there are inherent limitations in establishing the safety and efficacy of the correction by extrapolating from mouse models to humans. Several developments have allowed more ‘human’ versions of the disease to be studied at a level of detail not possible in sickle cell patients. Principally, the development of erythroid differentiation protocols for human CD34^+^ cells allows for mature red blood cells to be produced *in vitro*, and then behave normally upon infusion into HSC donors [21,22]. This technique introduces alternative and supporting tests for gene therapy methods, the potential for patient-specific approaches, and the exploration of rare or confounding disorders of red blood cells which may lack suitable animal models [23]. The further development of small-scale electroporation technology could allow a pilot gene therapy study to use CD34^+^ cells harvested from non-mobilized peripheral blood from patients.

Previously, we reported the use of *SB* to deliver an erythroid-specific IHK-driven hybrid gene to express β-globin in erythroid cell lines and the mature erythroid progeny of transduced CD34^+^ cells from normal donors [24,25]. The 1 kb erythroid promoter IHK can provide high-level expression of β-globin in hematopoietic cells and is composed of the *ALAS2* intron 8 strong erythroid enhancer, the HS-40 core element upstream from the ζ-globin gene, and the *Ankryin-1* promoter [26]. Fetal hemoglobin and modified hemoglobins have a greater ability to prevent sickling pathology than native β-globin. In addition the modified β-globin derivative β^T87Q^, which has anti-sickling properties, has been used in an ongoing human trial for β-thalassemia as well as in this study to maximize potential benefits of an *SB* delivered IHK transgene [12,27]. In this study, we show how mature red blood cells derived from β^S^/β^S^ CD34^+^ cells display the characteristic sickling morphology upon deoxygenation with metabisulfite and the ability of IHK-β^T87Q^-globin to improve this measure of disease pathophysiology. We have adapted imaging cytometry and videography as methods of assessing this phenomenon. These results show the potential clinical utility of IHK-β-globin-based gene therapy for SCA and a novel method for studying changes in red blood cell morphology in response to gene therapy.

## Results

### Nucleofected CD34+ cells from β^S^/β^S^ bone marrow differentiate into mature cultured red blood cells (cRBCs)

**Figure 1:**
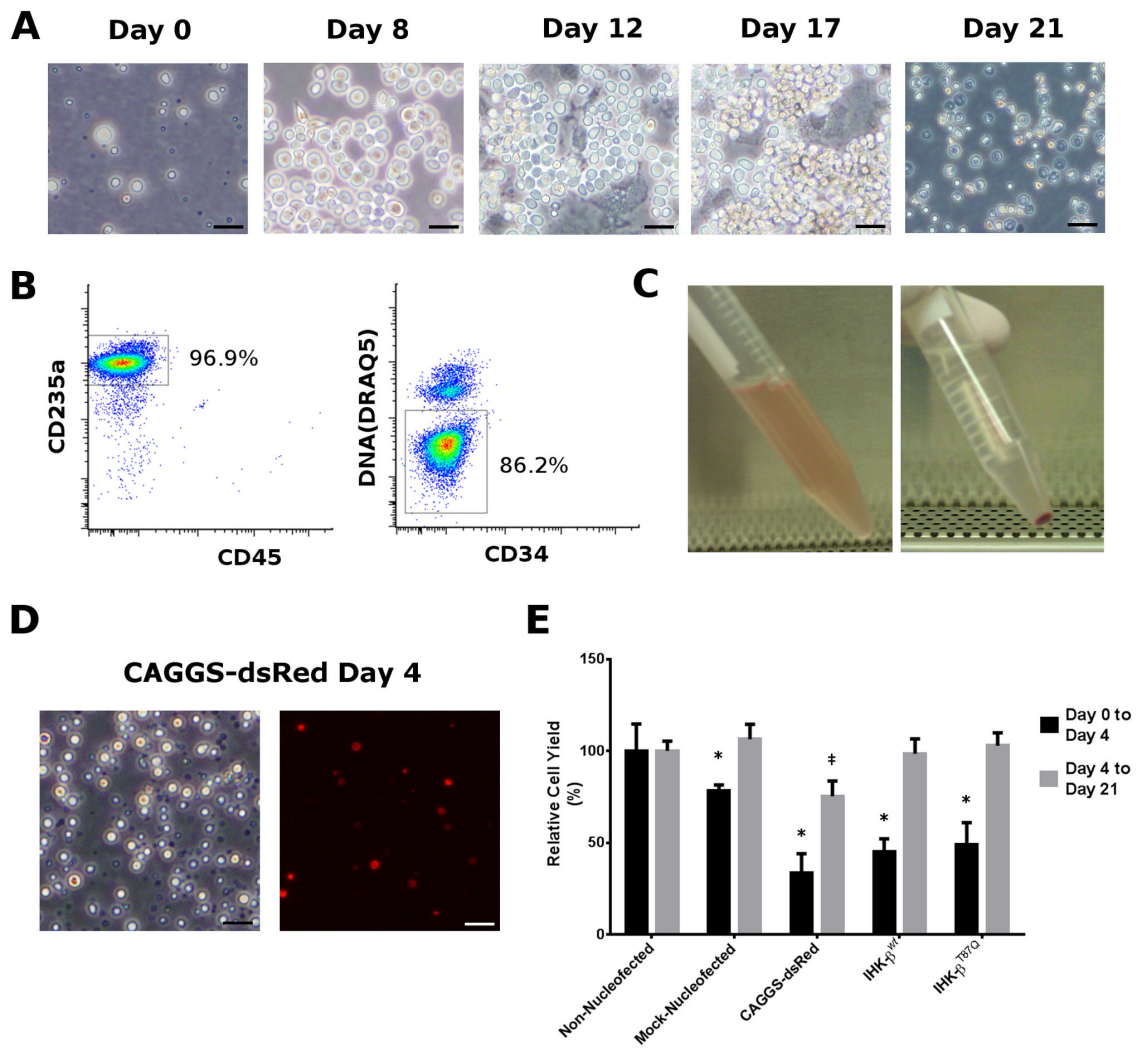
Erythroid culture and differentiation. (**A**) Fields from an IHK-β^T87Q^-globin treated β^S^/β^S^ CD34+ differentiation at day 0 (post-nucleofection), day 8, day 12 (on feeder layer), day 17 (on feeder layer), and day 21 (washed and replated without a feeder layer prior to analysis). All images were captured at 300X magnification, scale bar = 20 μm. (**B**) Flow cytometry of the same trial as panel A at day 21 using antibodies specific for CD235a, CD45, CD34, and DNA. Both panels depict the same singlet-gated population. (**C**) Approximately 3x10^7^ non-adherent cRBCs at day 21 suspended in PBS at an approximate hematocrit of 0.1% (left) and as a pellet (right). (**D**) pKT2/meIF-SB100X//CAGGS-dsRed nucleofected cells at day 4 in brightfield (left panel), and dsRed epifluorescence (right panel). Images at 300X magnification, scale bars = 20 μm. (**E**) Cell yields relative to non-nucleofected cells from day 0 to day 4 and from day 4 to day 21. * indicates *p* < 0.05 day 0 to day 4, students t-test; ‡ indicates *p* < 0.05 day 4 to day 21, students t-test, error bars shown as mean ± SD.

Differentiation trials for controls, IHK β-globin and β^T87Q^-globin transduced cells showed successful differentiation over 21 days to enucleated red blood cells (Figure 1A, B, C). Cells progressed through an initial expansion, to an adherent hemoglobinizing phase, and to non-adherent enucleated phase when they became mature cRBCs (Figure 1A, Days 0 and 8, Days 12 and 17, and Day 21, respectively). These cells were > 95% CD235^+^ and > 75% enucleated, with less than 1% residual CD45^+^ or CD34^+^ in all trials (Figure 1B, β ^T87Q^-globin). There were no significant differences between the β^S^/β^S^ CD34^+^ trials in CD235 expression or level of enucleation (data not shown). Cells nucleofected with a dsRed transposon did express the transgene at 17.0 ± 8.4% at day 4 (Figure 1D, single trial shown); however, no dsRed positive cRBCs were observed at day 21 (data not shown). There were significant differences in cell yields at 4 days in all nucleofection treatment conditions when normalized to β^S^/β^S^ non-nucleofected controls (100.0 ± 14.7%). Mock-nucleofected yielded 78.5 ± 3.0%; dsRed 33.7 ± 10.4%; β-globin 45.1 ± 7.1%; and β^T87Q^-globin 49.2 ± 12.1% (Figure 1E, *p* < 0.05). DsRed trials, however, were the only trials to show a significantly diminished cell yield from day 4 to day 21, (79.0 ± 8.1%, *p* < 0.05).

### IHK transgene is retained and produces therapeutic globin chains in nucleofected cells

**Figure 2:**
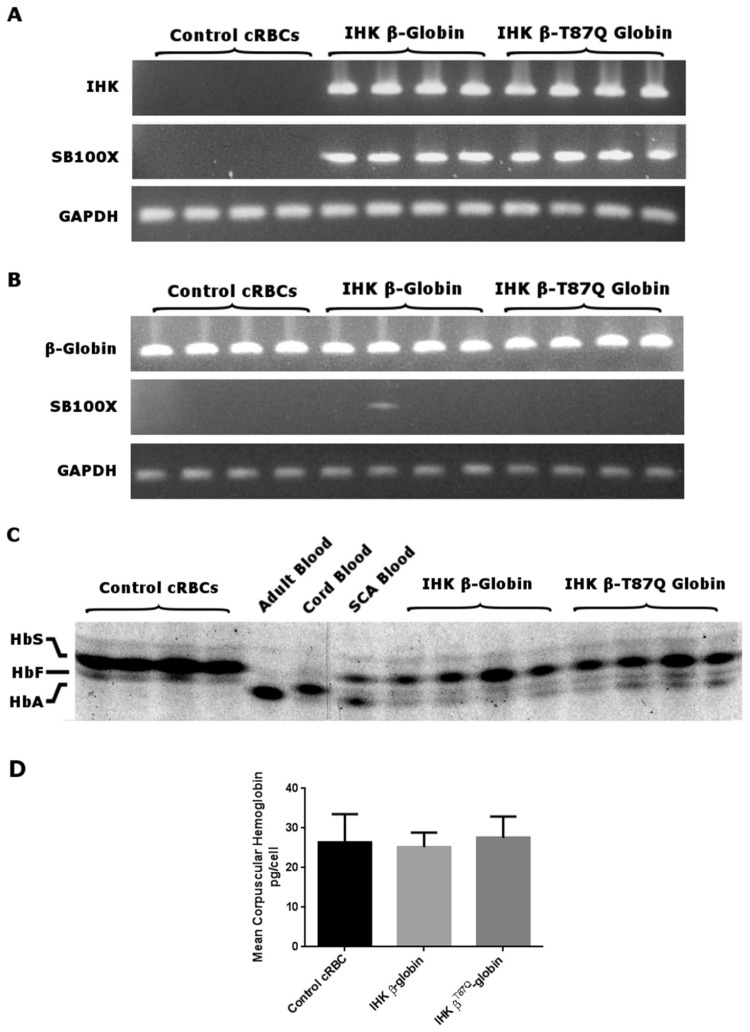
Molecular analysis of IHK-treated cells. (**A**) 100 ng of total genomic DNA isolated 15 days after nucleofection of four mock, four IHK-β-globin, and four IHK-β^T87Q^-globin groups was PCR-amplified to detect the IHK transgene, the SB100X coding sequence, and endogenous GAPDH DNA. (**B**) 50 ng of total RNA from four mock, four IHK-β-globin, and four IHK-β^T87Q^-globin nucleofected groups was RT-PCR-amplified to detect β-globin, SB100X, and GAPDH mRNAs. (**C**) 10 μg of total Hb from cRBC hypotonic lysate was resolved using native isoelectric focusing and stained with Coomassie Brilliant Blue. Control samples for HbA were from normal adult RBCs; HbF was from normal cord blood RBCs; and HbS/HbA was from a SCA patient on exchange transfusion with approximately 40% HbA. β^T87Q^- and β-globin containing Hb are not distinguishable in this assay by their isoelectric points. (**D**) Lysate volume, concentration, and input cell numbers for panel C were used to calculate MCH in pg/cell. There were no significant differences between trials; data shown as mean ± SD.

Cells from 4 separate nucleofections were analyzed for transgene retention 15 days after nucleofection, prior to enucleation, and were found to contain IHK-β-globin DNA and in one instance, a randomly integrated SB100X transposase DNA (Figure 2A), expressing trace levels of mRNA. Total β-globin mRNA was robustly expressed in all trials and the addition of the IHK β-globin transgenes did not appreciably affect total β-globin RNA levels (Figure 2B). Flow cytometric analysis of the trial expressing trace SB100X mRNA showed no differences in CD34, CD45, CD235a, and DNA content (data not shown). Isoelectric focusing analysis, showed the production of IHK-expressed normal β-globin and β^T87Q^-globin bearing hemoglobin-A (HbA) in all the treated groups (Figure 2C). Gel quantitation showed IHK-β-globin trials had 4.1 ± 0.8% HbA and β^T87Q^-globin trials showed 6.6 ± 1.3% HbA as percent of overall Hb, which was absent in untreated controls. Treated cells did not show appreciable differences in relative hemoglobin-F (HbF) expression from controls. Mean corpuscular hemoglobin (MCH) concentrations was calculated, and there were no significant differences between trials with 26.3 ± 7.2 pg/cell in controls, 25.2 ± 3.7 pg/cell in β-globin, and 27.5 ± 5.3 pg/cell in β^T87Q^-globin (Figure 2D).

### Treated cRBCs have improved sickling kinetics and endpoints

**Figure 3:**
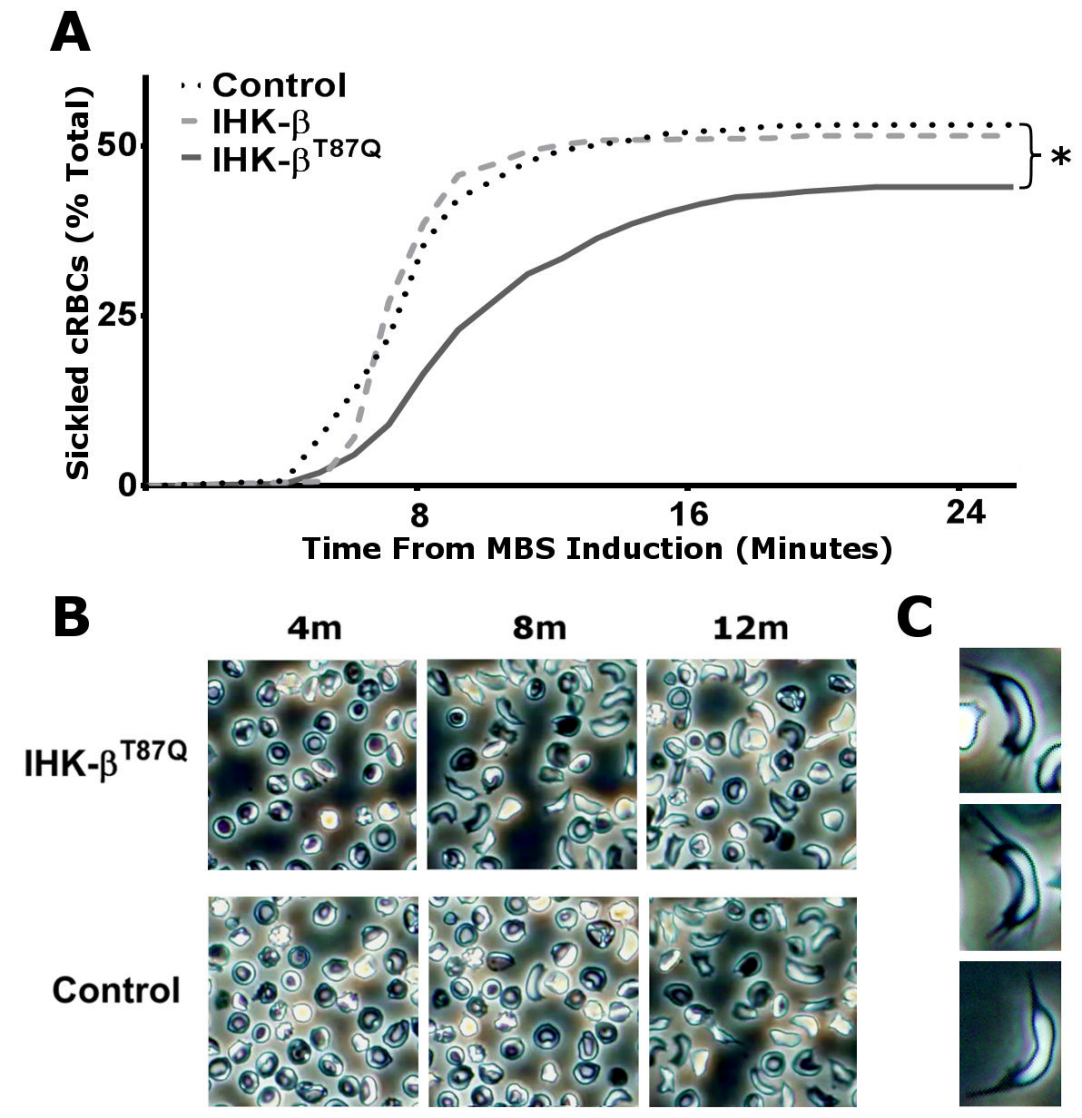
Video analysis of cRBC sickling. (**A**) Video at 300X magnification of sodium metabisulfite (MBS)-induced sickling of cRBCs from four separate mock (Control), and two IHK-β-globin, or four IHK-β^T87Q^-globin nucleofections. Sickling was analyzed at 256 frame intervals with a cell scoring positive for a rapid morphologic change from the previous frame; and each cell treated as a separate event for a total of 568 cells in the control (black dotted line), 310 cells in the IHK-β-globin treated trials (light grey dashed line), and 609 cells in the IHK-β^T87Q^-globin treated trials (dark grey solid line). Data shown as mean percent sickled, * *p* < 0.01, Gehan-Breslow-Wilcoxon test. (**B**) Representative frames from one IHK-β^T87Q^-globin treated field and one control field at 4, 8, and 12 min following MBS induction. (**C**) Spiculated cRBC examples from MBS-induced controls.

Video microscopy of cRBCs induced to sickle showed a significant decrease in percent of sickled cells during the 30 min MBS-induced video assay period in control versus IHK-β^T87Q^-globin treated cells from 53.2 ± 2.2% to 43.9 ± 2.0%, respectively (*p* < 0.001, Gehan-Wilcoxon-Breslow Test) (Figure 3A). Additionally, for those cells that did sickle, the median time to sickling was significantly different with 8.5 min for controls and 9.6 min in the IHK-β^T87Q^-globin treated cells (Wilcoxon Test, *p* < 0.001). For both sets, the maximum rate of sickling decreased from 2.3 x 10^-3^ sickling cells/total cells/sec in the control trials to 1.26 x 10^-3^ sickling cells/total cells/sec in the IHK-β^T87Q^-globin treated trials. IHK-β-globin treated cells did not show significant differences from controls, with 51.4 ± 2.8% sickling during the assay period with an 8.6 min median time to sickling and a peak sickling rate of 3.1 x 10^-3^ sickling cells/total cells/sec (Figure 3A). In addition, there was an absolute delay time of 4.3 min before any of the cells sickled. Video S1 and Video S2 of control and IHK-β^T87Q^-globin treated cells, respectively, show how cells undergoing sickling did so rapidly, and typically within a few seconds of the beginning of morphologic changes, consistent with rapid Hb polymerization (Figure 3B). In addition to the gross sickling morphology, spiculation was clearly observable with fibers protruding from the membranes in subsets of sickled cells in all trials (Figure 3C, control shown). In response to 5% O_2_ - 95% N_2_ we found that cells which were exposed to room air during culture did not show sickling or other gross morphological changes (data not shown). This was, in fact, consistent with previous reports [28,29].

### Sickling and non-sickling cRBCs are statistically separable with imaging cytometry

**Figure 4:**
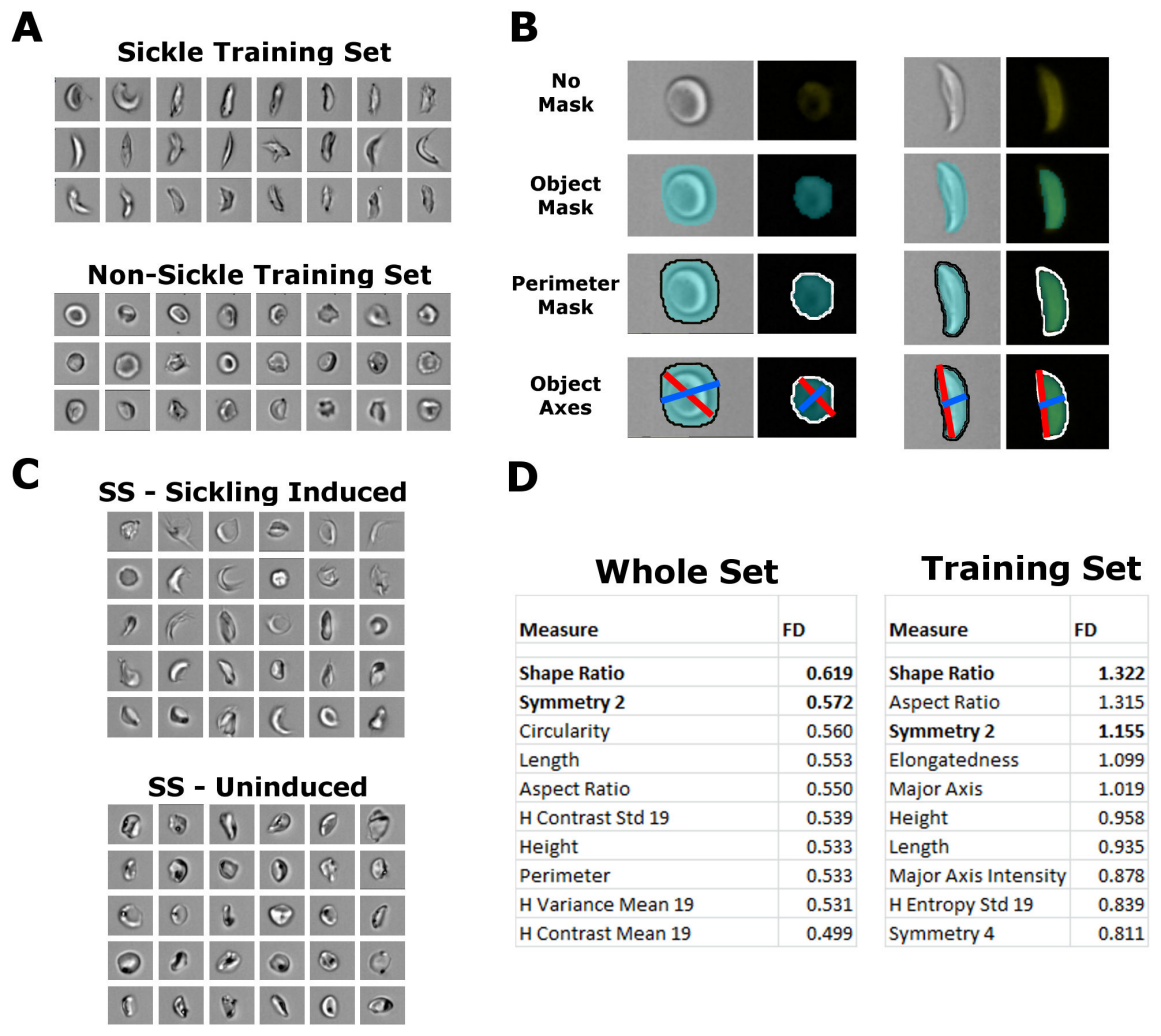
Imaging cytometry scoring of sickling. (**A**) Examples of cells selected by hand as examples of ‘sickle’ or ‘non-sickle’ for training sets. (**B**) Object masks (as defined by IDEAS software) and representations of perimeter and object axis metrics used by the software to quantify cell morphology. (**C**) Examples of single cells from an β^S^/β^S^ trial with and without induced sickling and used for statistical comparison (**D**) The 10 highest Fisher’s discriminant (FD) of means values as calculated by IDEAS for software-defined measures. Bold indicates measure used in combined analysis. Data shown from separate MBS-induced SS controls and a matched uninduced SS control.

Control cRBCs were induced with metabisulfite and fixed and stained for analysis by imaging cytometry. Gating was set to isolate single enucleated cRBCs for comparison and analysis (Figure S1). Nucleated cells and expelled nuclei, debris and aggregates, out-of-focus objects, and CD235^-^ cells were excluded from further analysis leaving mature cRBCs (Figure S1, lower right set). To identify the optimal metrics for separating cells based on gross sickling morphology, example cells were selected by hand for statistical comparison (Figure 4A). These sets were measured and compared using some of the hundreds of possible parameters available in the software, which primarily relies on masks, or image subsets to determine information such as size, shape, and brightness of the object (Figure 4B). To further validate and refine potential metrics, we compared β^S^/β^S^ cRBCs before and after sickling induction without hand selection, using 10^4^ cells from each condition for comparison (Figure 4C). The ability of a metric to separate the sickle from non-sickle training sets and the induced and uninduced data sets were then ranked by Fisher’s discriminant for optimization (Figure 4D). The highest scoring metrics were related to ratios of axes and dimensions, the highest being ‘Shape Ratio’, measures of symmetry of the cells, measures of minimum and maximum dimensions and several different measures related to the local variations in texture, such as “H Contrast” and “H Entropy”. Alternative analyses on star-shaped and wrinkled cells, which may be potential alternative outcomes of Hb polymerization, showed that these training sets could not be well-separated within trials or between trials of β^S^/β^S^ cRBCs and β^wt^/β^wt^ cRBCs (highest Fisher’s discriminant = 0.58, β^S^/β^S^ intratrial). Because cells can pass through the detector in any orientation, hand-picked sickled cRBCs were compared to hand-picked ‘sideways’ or non-discoid cRBCs in uninduced trials. The highest metric of separation was ‘Length’ (Fd = 1.61), with ‘Shape Ratio’ (Fd = 1.21) and ‘Symmetry 2’ (Fd = 0.91) also showing reasonable separation. In this way 2-fold Symmetry, or ‘Symmetry 2’, was higher in sickled cells while the minimum thickness divided by the length, or ‘Shape Ratio’, decreased in sickled cells. These metrics were used to derive a ‘Sickle Score’, which was used in further analyses and calculated as ‘Symmetry 2’ divided by ‘Shape Ratio’ from the brightfield channel. The combined metric had a Fisher’s discriminant of 1.71, versus 1.32 for ‘Shape Ratio’ alone.

### Treated cRBCs show decreased sickling with imaging cytometry

**Figure 5:**
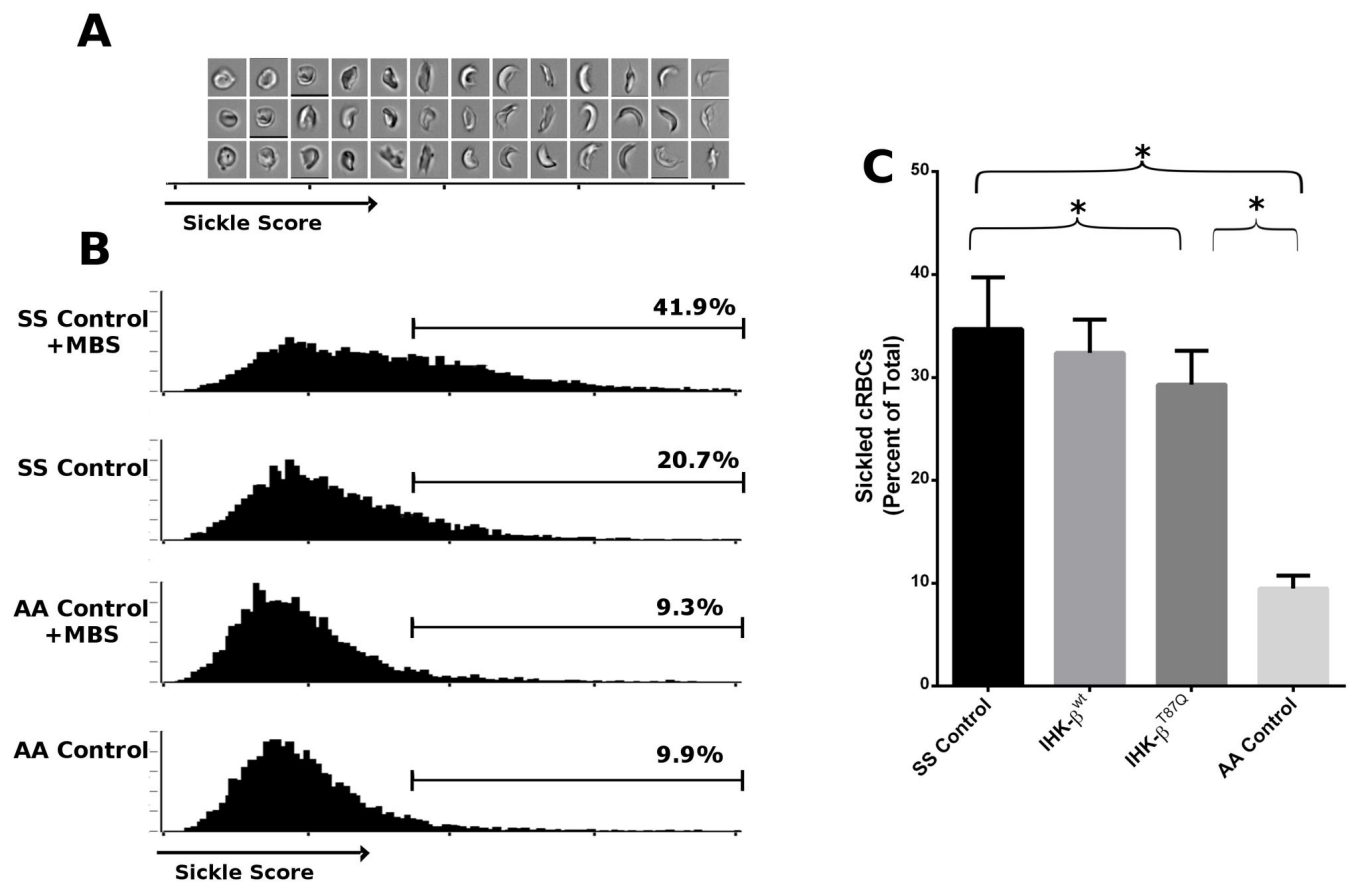
Imaging cytometry of cRBC sickling. (**A**) Images from ImageStream of an MBS-induced SS control trial ordered by sickle score and corresponding to histogram data. (**B**) Histogram and gating of an β^S^/β^S^ CD34^+^ (SS) control trial with and without MBS induction and a β^wt^/β^wt^ CD34^+^ (AA) with and without MBS induction. Gates were set for ^~^ 10% spillover in AA trials. (**C**) Percent of mature cRBCs scoring positive for sickled shape distortion from β^S^/β^S^ cRBCs from seven mock nucleofected trials (SS Control), six IHK-β^wt^-globin, six IHK-β^T87Q^-globin, and four β^wt^/β^wt^ mock nucleofected groups (AA control). Data shown as mean ± SD, * *p* < 0.05, students t test. AA controls were statistically significant from all SS trials, *p* < 0.05, students t test.

Fixed cRBCs induced with metabisulfite from treated trials and controls were analyzed using imaging cytometry and demonstrated a range of scores on the software-calculated metric of sickle shape distortion (Figure 5A). Sickling was induced in SS cRBCs with metabisulfite, but not in AA cRBCs (Figure 5B, representative controls). IHK-β^T87Q^-globin treated CD34^+^ cells showed a statistically significant decrease in the percent of cells gated positive for sickling from 34.8 ± 4.5% in the SS Controls to 29.5 ± 3.0% (*p* < 0.05, student t-test) (Figure 5C). IHK-β^wt^-globin treated CD34^+^ cells also showed a reduction in sickling to 32.4 ± 2.6%, which was not statistically significant (*p* = 0.30), but the trend was positive.

## Discussion

The therapeutic modification of hematopoietic stem cells is a natural target for correcting red blood cell pathologies. In this study, we have used hematopoietic stem cells from a SCA patient as a potential way to model gene therapy for the disease. We showed that CD34^+^ cells can be differentiated into fully mature enucleated erythrocytes *in vitro* and that these cells can be reproducibly induced to sickle with metabisulfite. While cells would sickle readily in response to metabisulfite, cells did not sickle in response to low oxygen tension alone. Similar results have been reported previously, including the observation that not all input RBCs will sickle in response to metabisulfite, and molecular results showed the presence of hemoglobin-S (HbS) as the major component of total Hb in cRBCs [28,29].

Our studies showed the characteristic sickling shape and spiculation characteristic of Hb polymerization. This model also showed the delay-time phenomenon and could potentially illustrate the increased sensitivity of the system to temperature, Hb concentration, and other deoxygenating conditions. The differentiation method does not appear to induce appreciable levels of non-adult Hbs in bone marrow cultures, which makes it intriguing to study numerous avenues of *in vitro* or *in vivo* investigations into SCA. Recent studies have shown significant genetic correction of SCA in iPS-derived erythroid cells, but did not report the sickling phenotype [30,31]. Another study showed terminal differentiation of cRBCs from iPS cells derived from SCA patients that did not sickle in response to deoxygenation. Of note, metabisulfite induction was not performed and the cells also retained significant non-adult Hbs during *in vitro* testing [32]. A phenotypic model of the disease process can provide important data about the biological significance of globin chain expression for approaches using gene therapy. Similar models for other RBC pathologies such as thalassemia, G6PD-deficiency and hereditary spherocytosis could utilize *in vitro* differentiation and analysis to provide improved tools for optimizing and evaluating potential therapies in patient-derived models.

CD34^+^ cells are robust and can be manipulated to express genes of interest. The cells also tolerated nucleofection and produced fully matured progeny, which harbored the therapeutic transgene, in this case a modified globin expressing vector. The cRBCs derived from CD34^+^ cells treated with these *SB* vectors showed expression of the therapeutic Hb. The IHK-β^T87Q^-globin-treated cells showed significantly reduced sickling as measured by both video scoring and scoring using imaging cytometry. This is in contrast to the IHK-β^wt^ treated trials, which expressed β-globin at a similar concentration as the modified globin, but did not reach a statistically significant improvement in sickling metrics. Indeed, the IHK-β^wt^ serves as an ideal control for the effects of IHK-β^T87Q^ transgene, utilizing the same delivery system while differing in only one amino acid, and highlights the biological differences in response to a gene therapy that can be demonstrated with an *in vitro* model of sickling. The comparison between the two IHK vectors supports the use of modified globins for gene therapies both for their anti-sickling properties, and their traceability [33,34]. We also observed that dsRed expressing vector treatment did not show persistent dsRed expression and had a deleterious effect on the maturation of cells in this system. While these analyses showed a difference in the classically elongated and curved sickled cRBCs, we were limited in our analyses of less distorted star-shaped and wrinkled types of cells from Hb polymerization. Thus, our metrics may have resulted in a potentially significant underestimation of Hb polymerization in these analyses [28,29].

The video- and machine-scored metrics used in this study were in overall agreement with each other showing an average of 53% control sickling on video and 35% sickling after fixation and machine scoring with similar sizes in treatment groups. Both methods indicated ^~^ 15-20% of the progeny of the modified HSCs had sufficient transgene expression to prevent or diminish their sickling. However, machine scoring should take into account the multiple modes of sickling and orientations in the flow cell when utilizing imaging cytometry. We found this difficult to incorporate in our analyses and it remains an inherent limitation to this imaging process. The two methods differ in their degree of background, with machine scoring unable to score as accurately as a human observer, as evidenced by spillover in the AA and SS uninduced trials during imaging cytometry, which did not occur in the video analysis. The higher proportion of cells sickling under direct observation compared with fixation and machine scoring likely indicates some degree of reversal of sickling or artifacts during the fixation process. Further use and refinement of machine scoring for sickling, while useful in this context for showing relative differences between large populations, requires confirmation by other methods and while promising is not yet superior to blinded human scoring.

The observed increase in HbA expression to 6% in the IHK-β^T87Q^-globin treated cells approached the level of 10% HbF induction needed to resolve much of the SCA pathology in a mouse model study [19]. The potential for selective advantage of corrected cells *in vivo* is an important consideration for a successful autologous non-ablative transplant where not all the HSCs can be corrected and/or selected. Potential pre-clinical applications of this system are many, especially given the relativly small amounts of cells needed for nucleofection. The ability to generate data from many patients in *in vitro* trials may be useful for understanding safety and efficacy and optimizing the preclinical gene therapy process. A particular advantage of an *in vitro* model is the ability to examine a more uniformly aged population of stem-cell progeny and to collect meaningful time series data of the consequences of a gene therapy intervention of cell development and expression. The ability in real time to examine the relationship between therapeutic globin expression and sickling will undoubtedly provide important information in considering a gene therapy approach for SCA and potentially other diseases.

 The successful introduction of a *SB* therapeutic transgene into CD34^+^ cells from an SCA patient and *in vitro* evidence of its ability to improve the sickling characteristics of these cells are important milestones in developing this non-viral gene therapy system. These results demonstrate the potential efficacy of the *SB* system clinically for gene therapy of SCA and the value of a patient-derived disease model for investigating avenues of treatment.

## Methods

### Ethics Statement

Umbilical cord blood was obtained through the National Cord Blood Donation Program in accordance with University of Minnesota Institutional Review Board approval and the declaration of Helsinki. Human homozygous β^S^/β^S^ bone marrow was obtained from a bone marrow transplant backup unit from a deceased patient in accordance with University of Minnesota Institutional Review Board approval and the declaration of Helsinki. Donors, or their families in the case of deceased donors, gave written and informed consent for the use of tissues in this study. Consent forms and procedures were approved by the University of Minnesota Institutional Review Board.

### *Sleeping Beauty* transposon constructs

Plasmids pKT2/meIF-SB100X//CAGGS-dsRed and pKT2/meIF-SB100X//IHK-β-Globin were constructed with standard molecular cloning techniques as previously described [24]. pKT2/meIF-SB100X//IHK-β^T87Q^-Globin was derived from IHK-β-Globin using mutagenic primers and the QuickChange II site-directed mutagenesis kit (Stratagene) [27]. Plasmids for nucleofection were purified using Endofree Plasmid Maxi kits (Qiagen).

### CD34^+^ cell purification

β^S^/β^S^ CD34^+^ cells were purified from cryopreserved bone marrow using the CliniMACS CD34^+^ (Miltenyi Biotechnology) magnetic selection system according to the manufacturer’s recommended protocol, and aliquots were cryopreserved and stored in liquid nitrogen. CD34^+^ purity was assessed prior to freezing using Anti-CD45 V500 (BD Biosciences) and anti-CD34-Alexa647 (eBioscience) and was > 90%. Normal adult bone marrow CD34^+^ selected cells were obtained commercially through Lonza.

### Nucleofection of CD34^+^ cells

Frozen CD34^+^ cells were thawed and incubated for 2 hr at < 5 x 10^5^ cell/mL in StemLine II Expansion Media (Sigma) supplemented with 100 ng/mL each of human stem cell factor (SCF), thrombopoietin (TPO), Flt-3 ligand (FLT3), and IL-6 (all R & D Systems). Cells were nucleofected with 2 μg of plasmid DNA using the 4D Nucleofector platform with the P3 Primary Cell Kit S (both Lonza) using 5 x 10^4^ cells per reaction as recommended by the manufacturer. Cells were recovered in 1 mL of pre-warmed erythroid differentiation media.

### Erythroid differentiation

Erythroid differentiation was performed as previously described [21]. Basal media was formulated using IMDM without glutamine and antibiotics (BioChrom AM), pre-screened lots of BSA (Stemcell Technologies) and Glutamax (Invitrogen). Briefly, cultures were initiated with < 5x10^4^ cells/mL and cultured for 8 days in basal media supplemented with 100 ng/mL SCF, 5ng/mL IL-3, 3U/mL EPO (R & D Systems) and 10^-6^ M HC. From days 8 to 11 cells were co-cultured on MS-5 feeder cells in basal media supplemented with 3U/mL EPO; from days 11 to 16 cells were co-cultured on MS-5 feeder cells in media supplemented with 2% heat inactivated (HI) AB male serum (Sigma); and from days 16 to 21 cells non-adherent cells were co-cultured on a fresh MS-5 feeder layer in media supplemented with 10% HI AB serum (Figure 1A).

### Sickling induction

Mature cultured red blood cells (cRBC) were washed and resuspended in basal media. Sickling was induced with either the addition of 1 volume of freshly prepared 2% sodium metabisulfite solution followed by incubation at 37°C or room temperature as indicated or by hypoxia using equilibration with 5% O_2_ - 95% N_2_. Cells were fixed in vacutainers with 5 volumes of freshly prepared and equilibrated 1% glutaraldehyde in 0.1M sodium cacodylate buffer, pH 7.4, as previously described and left overnight to preserve morphology [35].

### PCR analyses

DNA and RNA from nucleated cRBCs were collected at 14 days after initiation using the AllPrep Micro kit as recommended by the manufacturer (Qiagen). PCR analysis was performed using primers specific for the IHK Promoter, human β-globin, SB100X, and GAPDH [24]. Briefly, 100 ng of genomic DNA was subjected to PCR amplification for the IHK promoter, SB100X coding sequence, and the GAPDH gene using the Kapa HotStart PCR System. 50 ng of total RNA was subjected to PCR amplification for β-globin, SB100X, and GAPDH using the Titan OneTube RT-PCR kit.

### Isoelectric focusing

To collect Hb, mature cRBCs were lysed in 50 μL of water and centrifuged at *g* x 10^4^. Cleared lysate was quantitated for total Hb using Drabkin’s method at 540 nm. 10 μg of total Hb was resolved using ReadyGel pH 3-10 isoelectric gels (BioRad) and stained with coomassie blue as recommended by the manufacturer and imaged using an AlphaImager with transillumination (Alpha Innotech). Gel quantification was performed using FIJI software densitometry (Fiji.sc) with Integral OD without background correction, as previously described [36].

### Microscopy

Live and fixed cell images and video were acquired using an Olympus IX70 epifluorescence microscope with an Olympus DP71 CCD Camera. Video acquisition was performed at room temperature and air in 35 mm culture dishes at 4 frames per second during and following the addition of one volume of 2% sodium metabisulfite. Video was recorded for 30 min per trial. Video editing and frame extraction was performed using Avidemux 2.6. Extracted frames were overlayed with a grid and individual cells were scored in a blinded manner for morphologic changes from the previous frame.

### Flow cytometry

Cytometric data was acquired for at least ten thousand events using either an LSRII flow cytometer (BD Biosciences) or an Imagestream IS100 imaging cytometer (Amnis Corporation) and analyzed using FlowJo 7.6.5 software (TreeStar) or IDEAS Version 4 software (Amnis Corporation), respectively. cRBC were analyzed at 21 days post-nucleofection. Data were analyzed in template batches.

### Statistics

Statistical analysis of video, gel data, and cytometry data sets was performed using Graphpad PRISM 6 (Graphpad Software). Fisher’s discriminant analysis was performed using IDEAS 4.0.

## Supporting Information

Figure S1 **Imaging cytometry RBC gating.** Sequential gating and examples from imaging cytometry of an MBS-induced SS control. Histograms show events selected sequentially for a lack of DNA content as assessed by DRAQ5 staining, (‘Non-nucleated’ gate), a size range excluding most debris and aggregates, (‘Non-debris’ gate), a focus level permitting better shape analysis, (‘Mid-focus’ gate), and CD235-positive staining (‘CD235 – FITC’ gate). Images arrays show six examples of cells excluded and included by the gate respectively, with brightfield, FITC fluorescence, and DRAQ fluorescence images of each example.
(TIF)

Video S1 **SS Control cRBCs.** Video of MBS-induced sickling in SS control trial run at 12X speed and cropped, decolorized and compressed from the original. The video spans from 4 min to 12 min following MBS induction.
(MP4)

Video S2 **SS IHK-β^T87Q^ cRBCs.** Video of MBS-induced sickling in SS IHK-β^T87Q^ trial run at 12X speed and cropped, decolorized and compressed from the original. The video spans from 4 min to 12 min following MBS induction.
(MP4)
