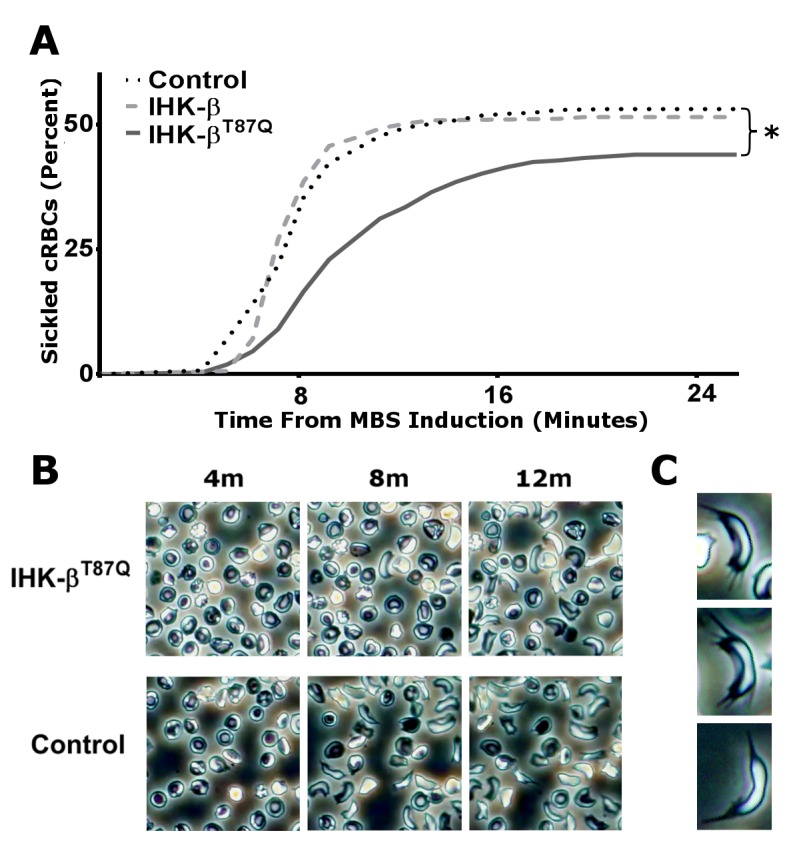# Correction: β-Globin *Sleeping Beauty* Transposon Reduces Red Blood Cell Sickling in a Patient-Derived CD34^+^-Based In Vitro Model

**DOI:** 10.1371/annotation/2b72460f-f6ba-4725-97cb-06e6dbd28b1a

**Published:** 2014-01-17

**Authors:** Lucas M. Sjeklocha, Phillip Y.-P. Wong, John D. Belcher, Gregory M. Vercellotti, Clifford J. Steer

In Figure 3B, the panels for 4m and 8m were erroneously switched with the lower row. Please see the corrected figure here: 

**Figure:**