# Supplementary figures and images for: β-Globin Sleeping Beauty Transposon Reduces Red Blood Cell Sickling in a Patient-Derived CD34+-Based In Vitro Model
Source: PLoS One. 2013 Nov 18;8(11):e80403. doi: 10.1371/journal.pone.0080403 (PMC3832362; doi:10.1371/journal.pone.0080403)

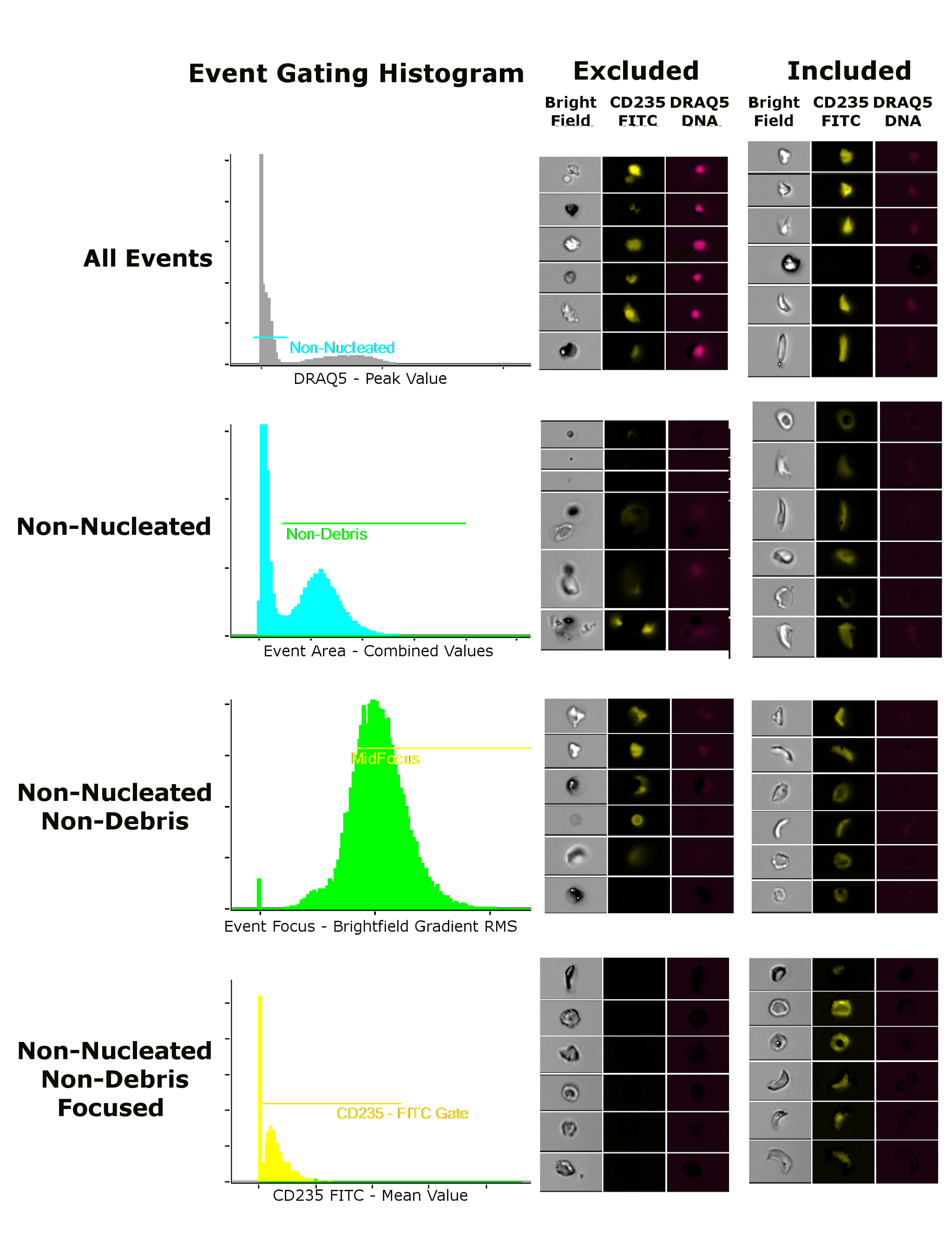

Supplement: Figure S1 — Imaging cytometry RBC gating. Sequential gating and examples from imaging cytometry of an MBS-induced SS control. Histograms show events selected sequentially for a lack of DNA content as assessed by DRAQ5 staining, (‘Non-nucleated’ gate), a size range excluding most debris and aggregates, (‘Non-debris’ gate), a focus level permitting better shape analysis, (‘Mid-focus’ gate), and CD235-positive staining (‘CD235 – FITC’ gate). Images arrays show six examples of cells excluded and included by the gate respectively, with brightfield, FITC fluorescence, and DRAQ fluorescence images of each example. (TIF) [file pone.0080403.s001.tif]
